# The Modified Scaled Adaptive Daqrouq Wavelet for Biomedical Non-Stationary Signals Analysis

**DOI:** 10.3390/s25175591

**Published:** 2025-09-08

**Authors:** Khaled Daqrouq, Rania A. Alharbey

**Affiliations:** 1 Department of Electrical and Computer Engineering, Engineering Faculty, King Abdulaziz University, Jeddah 21589, Saudi Arabia; kdaqrouq@kau.edu.sa; 2 Department of Mathematics, Faculty of Science, King Abdulaziz University, Jeddah 21589, Saudi Arabia

**Keywords:** continuous wavelet transform, modified scaled adaptive Daqrouq wavelet

## Abstract

The article presents Modified Scaled Adaptive Daqrouq Wavelet (MSADW) as an autonomous wavelet framework to overcome the analysis obstacles of traditional wavelets (Morlet and Daubechies) for signals with non-stationary characteristics. MSADW adjusts its waveform shape and frequency in real time based on the specific characteristics of the signal, allowing it to outperform conventional wavelet methods. The system reaches adaptability through three core methods featuring gradient-dependent scale adjustments for fast transient detection and smooth regions, and instantaneous frequency monitoring achieved by a combination of STFT and Hilbert transforms and an iterative error reduction process using gradient descent and genetic algorithms. Continuous Wavelet Transform (CWT) combined with Discrete Wavelet Transform (DWT) extracts features from ECG and speech signals. Throughout this process, MSADW maintains great time precision to detect transients as well as maintain sensitivity for the audio’s base stability. Testing MSADW in practical use reveals its superior performance because it detects R-peaks accurately within 0.01 s through zero-crossing methods, which combine P/T-wave detection with effective ECG signal segmentation and noise-free reconstructed speech ($\mathrm{MSE:}\;1.17\times 10^{-31}$). The localized parameterization framework of MSADW, enabled by feedback refinement, fulfills missing aspects in biomedical signal evaluation and creates space for low-cost real-time evaluation methods for medical devices and arrhythmia and ischemic detection platforms. The theoretical backbone for MSADW establishes itself because this work shows how wavelet analysis can transition toward managing non-stationary and noise-prone domains.

## 1. Introduction

Signal processing is transformed by implementing wavelet analysis to perform localized time-frequency analysis for complicated non-stationary signals such as ECGs and speech, as well as seismic data. Traditional wavelets implemented with Morlet, Daubechies, or Haar functions need fixed scale and frequency parameters as static elements that prevent them from reacting to changes in signal features [1]. Neither delicate timing details nor comprehensive low-frequency information can be simultaneously captured through traditional wavelet analysis because the fixed parameters force these combinations of approaches to compete against each other. These defects prevent medical diagnostic tools from working efficiently because they need accurate detection of brief events alongside reliable baseline detection [1,2].

This research adopts MSADW (Daqrouq), which functions as a self-optimizing wavelet framework to solve these weaknesses. MSADW creates real-time adjustments to its  σ(t) scale and ω(t) frequency together with waveform shape modifications, while it aligns with local signal characteristics. MSADW solves the time–frequency resolution conflict through an integrated system that evaluates gradient-scale parameters and tracks instantaneous frequencies while providing reconstruction feedback abilities, which yields unmatched performance capabilities. Through its implementation, MSADW addresses one of the most important gaps in biomedical signal analysis because it meets the requirements of detecting precise transients that operate in a noisy environment.

Biological signals require wavelet analysis because they demonstrate nonstationary patterns. The tools assist researchers in extracting key features as well as categorizing and segmenting vital signals originating from biological systems [3]. Data compression and biomedical imaging and diagnostic quality improvement occur through wavelet function applications. The time–frequency analysis framework that wavelets provide functions in one and two dimensions of signals and enables their robust evaluation [4,5]. Their application enables both noise reduction and data cleaning processes that produce essential conditions for statistical analysis accuracy [6].

Researchers maintain that traditional signal processing methods using Fourier transforms preserve their validity in particular applications because they maintain simplicity and established frameworks, although wavelet functions demonstrate significant advantages for signal processing. A technique exists for creating orthonormal wavelets that follow specific signals by using least squares optimization. Through this method, a scaling function obtains orthonormal multiresolution analysis (OMRA) generation properties according to [7]. The development of specialized wavelet functions for use in acoustic emission signals during fault diagnosis resulted in better performance than typical wavelet functions [8]. The continuous wavelet transform is particularly useful in analyzing non-stationary signals, especially in biomedical applications, thus providing improved understanding of time-dependent systems [8,9]. Wavelet space orthogonality and time window implementation improve non-stationary signal decomposition, which enhances both deconvolution and denoising methods [9]. Advanced Signal Processing Techniques GNWPT represents signal components by normalization, so it improves signal processing in nonlinear filtering and extraction from noise because it utilizes advanced masking techniques [10].

The study puts MSADW on a solid theoretical basis while showing how it can transform wavelet creation to customize signal analysis during real-time applications to noise. This adaptive framework redefines wavelet design, offering a change in basic assumptions toward signal-specific, resource-efficient analysis for biomedical and real-time applications.

## 2. Theoretical Framework

The Daqrouq wavelet family MSADW operates through a complex system that modifies the wavelet shape together with scale parameters and frequency specifications based on signal local attributes. The methodology stands in direct opposition to traditional wavelets because it uses adjustable wavelet shapes and unique scale parameters. The description below shows how MSADW, with its design, advantages, and disadvantages, stands apart from traditional wavelet analysis methods.

The MSADW technique is a new technique developed by the authors to enhance time–frequency resolution and flexibility in non-stationary signal processing. In contrast to classical wavelets, ADW provides increased localization and minimized redundancy of the basis functions design. More theoretical information and benchmarking will be studied next time.

### 2.1. Mathematical Framework for MSADW

**Figure 1:**
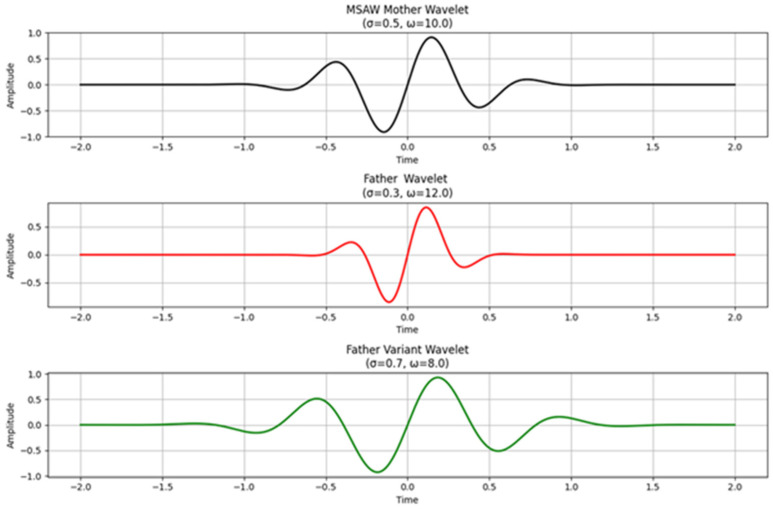
Mother wavelet, father wavelet, and an alternative father wavelet variant from the MSADW (Minimum Asymmetry) wavelet family.

The MSADW exists as a parameterized mathematical function (see Figure 1):

$$\psi_{MSAW}\left(t;\sigma \left(t\right),\omega \left(t\right)\right)=\underset{Localization}{\underbrace{Adaptive\;Envelope}}\cdot e^{-\frac{t^{2}}{\sigma {\left(t\right)}^{2}}}\cdot \underset{Frequency\;Modulation}{\underbrace{sin\left(\omega \left(t\right)t\right)}}$$

Where:

- Adaptive Envelope is an amplitude function that varies data-dependently, or is otherwise dynamically adjusted, to better adapt the overall shape of the wavelet to the morphology of the signal.
- The temporal version of the scale parameter σ(t) defines the localization width while showing reduced widths in high-frequency zones and larger widths near smooth zones.
- An adaptive frequency function ω(t) tracks the instantaneous signal frequencies during its operation.
- The signal fidelity optimization leads to an adaptive envelope of the wavelet form, which includes Gaussian and exponential.

### 2.2. Key Adaptive Mechanisms

#### Adaptive Envelope Definition

Mathematically, the adaptive envelope may be defined as:

A(t)=E(t)⋅ϕ(t)

where E(t) is the energy of the signal at time t, and ϕ(t) is the adaptive modulation function that adjusts based on local signal characteristics.

A. **Signal-Driven Scale Adaptation** $\sigma \left(t\right):$
  - The adjustment of $\sigma \left(t\right)$ depends on the signal variability that emerges locally.
  - Implementation:
$$\sigma \left(t\right)=\left\{\begin{array}{c} \sigma_{min},\;\;\mathrm{if}\;|\nabla_{s\left(t\right)}|>\theta_{high}\\ \sigma_{max},\;\;\;\mathrm{if}|\nabla_{s\left(t\right)}|<\theta_{low} \end{array}\right.$$
  Here, $\nabla_{s(t)}$ is the signal gradient, and $\theta_{high}$,${\;\theta }_{low}$ are thresholds for detecting abrupt changes or smooth regions.
  - Effect: The method provides both high temporal precision for detecting sudden shifts (e.g., ECG R-peaks) and data compression methods for stable periods.

Such dual terminology highlights both the functional role (time-varying scale) and adaptive behavior (response to signal content) of σ(t), and the two terms must be preserved in writing for clarity.

B. **Adaptive Frequency Modulation *ω*(*t*)**
  - The instantaneous frequency of a signal can be tracked by implementing Hilbert transforms or short-time Fourier transforms (STFT) techniques.
  - Frequency parameters ω(t):
    $$\omega \left(t\right)=\frac{d}{dt}arg\left\{\left[H[s\left(t\right)]\right]\right\},$$
  where H{s(t)} is the Hilbert transform of the signal *s*(*t*), over the time  t, and arg denoted for argument, the angle of the complex number presented by H[s(t)].
  - Effect: The mechanism keeps the wavelet in line with the oscillatory content of the signal by making continuous adjustments to be in line with the instantaneous frequency content of the signal.

C. **Optimization via Reconstruction Error Minimization**
  - Objective: Minimize the error between the original signal *s*(*t*) and its wavelet reconstruction:
    $$\underset{\sigma (t),\omega (t)}{\min}{∥s(t)-W^{-1}[W\{s(t)\}]∥}^{2},$$
    where W denotes the wavelet transform. The wavelet transform and the reconstructed signal are both dependent on the ω(t) since this determines the time-varying frequency of the wavelet basis functions with which it is performed through analysis and reconstruction.
  - Techniques: Gradient descent, genetic algorithms, or particle swarm optimization.

D. **Feedback-Driven Refinement**
  - Mechanism: Iteratively update σ(t) and ω(t) with a learning rate η, using residuals, for each iteration k:
    $$\sigma_{k+1}\left(t\right)=\sigma_{k}\left(t\right)+\eta \cdot \frac{\partial L}{\partial_{\sigma k}\left(t\right)},\omega_{k+1}\left(t\right)=\omega_{k}\left(t\right)+\eta \cdot \frac{\partial L}{\partial_{\omega k}(t)}$$
  - Loss Function L: Sparsity, entropy, or reconstruction accuracy.
  - Figure 1 shows a step-by-step algorithm of optimizing signal parameters within a wavelet analysis scheme. It is a sequence of colorful steps where each one of them corresponds to a vital stage of the optimization process.

### 2.3. Contrasts with Traditional Wavelets

**Figure 2:**
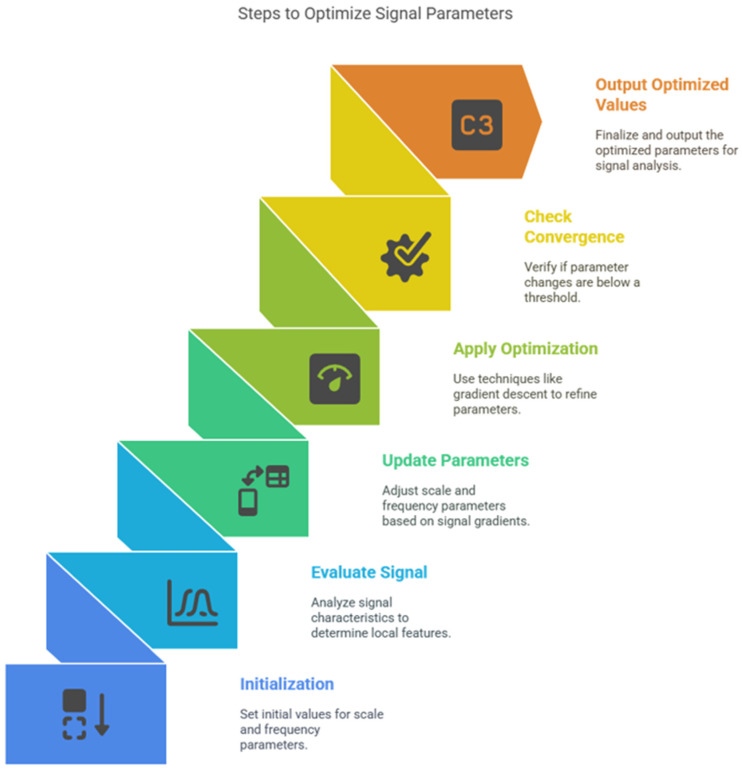
The flowchart of the optimization process.

**Figure 3:**
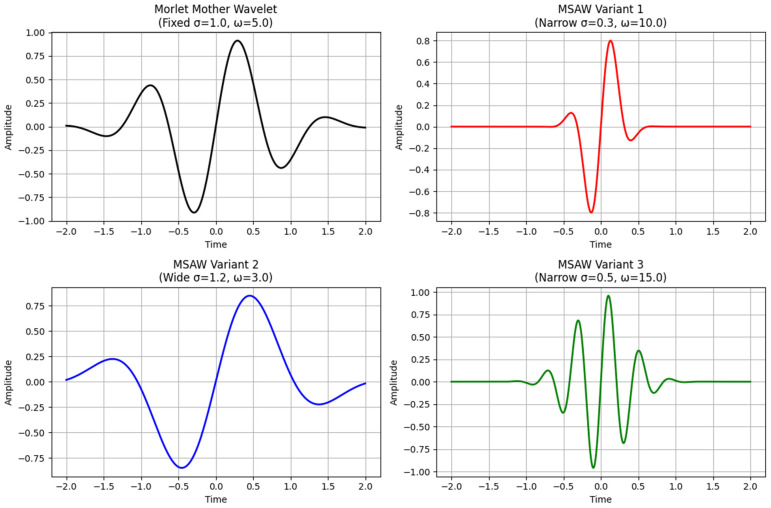
Illustration of different wavelets for comparison: The MSADW represents an adaptive signal analytical technique that opposes traditional techniques such as the Morlet wavelet (see Figure 1, Figure 3 and Figure 4, Table 1 and Table 2).

In Table 1 and Table 2, we compare the traditional wavelets with our proposed MSADW that can dynamically adjust the scale allowing better localization. The dynamic scale adjusment allow us to track instantaneous frequency changes propperly, as shown in Figure 1, Figure 2 and Figure 3. This adaptability enhances its effectiveness for signals with both transients and smooth regions. The MSADW differs from the Morlet wavelet because it modifies its waveform attributes while varying its characteristics depending on the signal’s local features. These adaptive abilities of MSADW through shape transformations and oscillatory patterns at different scales and frequency bands optimize the method for analyzing signals such as ECG, EEG, and speech, which are non-stationary. Traditional Daubechies or Morlet use a fixed family of parameters; there is flexibility in MSADW to customize the parameters. The adaptive and feedback processes in MSADW work to enhance the overall performance, but they would need additional computing resources since the increased processing time and complexity occur due to the introduction of optimization and feedback mechanisms.

**Figure 4:**
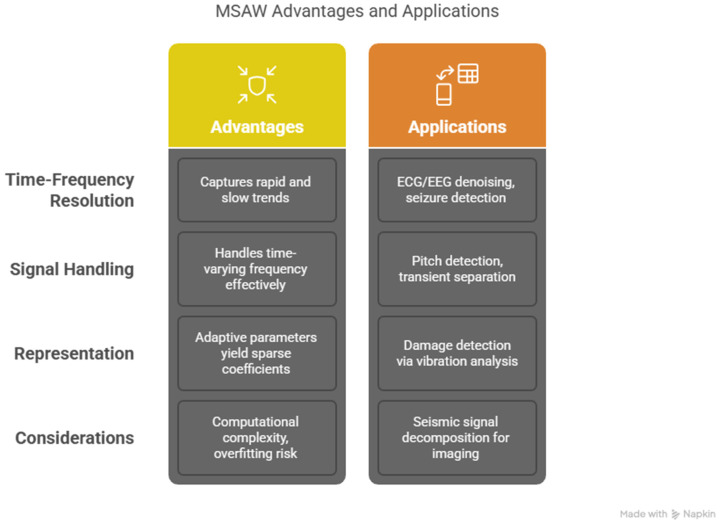
Advantages and applications of MSADW.

### 2.4. Challenges and Considerations

- The real-time adaptation process may need to use approximations because it operates on approximate values of σ(t) and ω(t).
- The process of optimization should strike a balance between how much it adapts and its ability to generalize the results to control noise amplification.
- Weak starting values for σ(t) and ω(t) parameters diminish the convergence rate.

The design of MSADW entails giving up orthogonality, orthonormality, and symmetry to develop adaptive signal analysis in real-time. The power of MSADW stems from solving non-stationary biomedical signals through its unique combination of localized parameterization control and feedback refinement, and detection from amplitude and zero-crossing measures. It supplements the current debate regarding the application of approximation, sensitivity towards convergence, and initialization problems.

## 3. Optimization Techniques Applications

### 3.1. Gradient Descent

**Figure 5:**
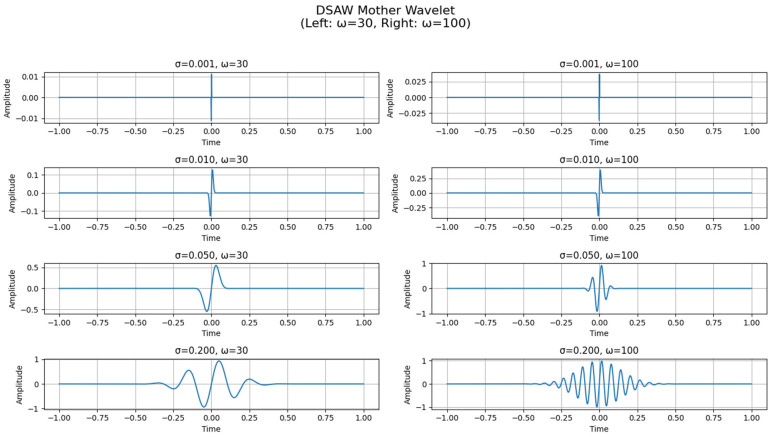
MSADW for different scales and different frequencies.

The purpose of minimizing the use of gradient descent is to reduce the reconstruction error between the original signal s(t) and its wavelet transform (see Figure 5). This is done in the following steps:

- Initialization: assign initial values to σ(t) and ω(t).
- Calculation of Error: At any one iteration, calculate the error.

$$E=∥s\left(t\right)-W^{-}1\left[W\left(s\left(t\right)\right)\right]{∥}^{2},$$

W is the wavelet transform.

- Parameter Update: parameters are updated with:

$$\sigma (t)\leftarrow \sigma (t)-\eta \frac{\partial E}{\partial \sigma (t)}$$

$$\omega (t)\leftarrow \omega (t)-\eta \frac{\partial E}{\partial \omega (t)}$$

where the learning rate is η.

This iteration is conducted until the convergence criteria are satisfied.

### 3.2. Genetic Algorithms

**Figure 6:**
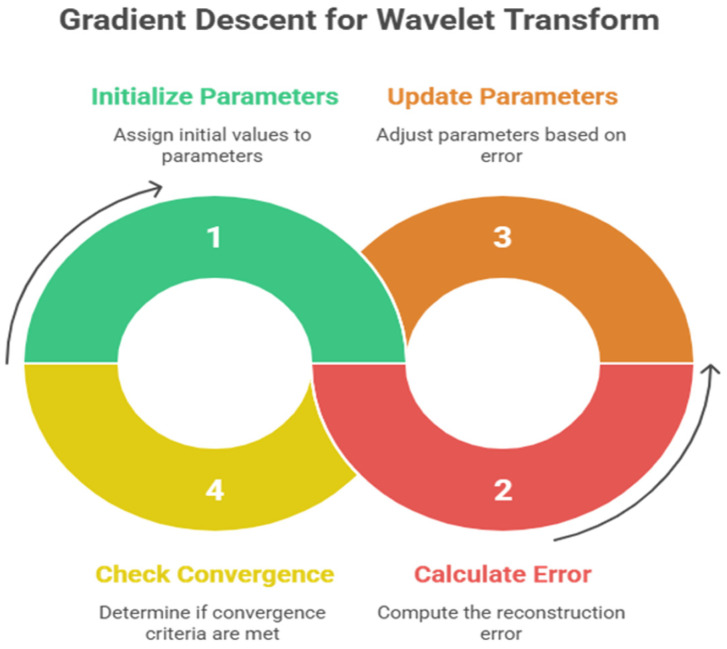
Gradient descent method for wavelet function optimization.

Genetic algorithms are used to optimize the parameters further by exploring a wider search space (see Figure 6). The method includes:

- Population Initialization: Generate an initial population of candidate solutions for σ(t) and ω(t).
- Fitness Evaluation: Evaluate the performance of each candidate according to the error measure E.
- Selection: Select the ones who were the highest performing to be spread into the next generation.
- Crossover and Mutation: Use the crossover and mutation operators to generate new candidate solutions, which introduces diversity in the candidate solutions.
- Iteration: Reassessment, reselection, and reuse of the genetic operations up to convergence of the optimization.
- Repeat: Again, evaluate, choose, and conduct the genetic operations until there is a balance in the optimization process.

The optimization algorithm that incorporates a gradient descent (fine-tuning) and genetic algorithms (exploration) produces more accuracy when studying the parameters of the signal.

## 4. Experimental Validation

**Figure 7:**
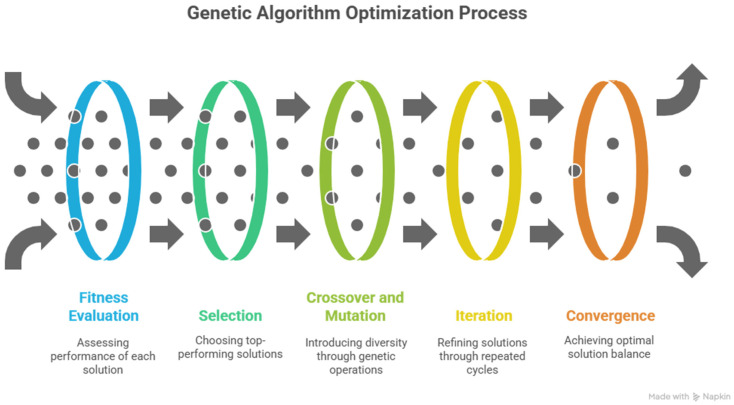
Genetic algorithm method for wavelet function optimization.

An image in Figure 7 demonstrates the DWT applied to a random speech signal through the MSADW function, which displays its effectiveness for speech signal processing. The figure presents the original speech signal and three levels of DWT approximation and detail coefficients. The multi-resolution analysis technique in DWT extracts vital low-frequency information together with high-frequency information required for tasks including speech recognition and enhancement as well as classification. The multiple detail coefficient levels generate essential data that lead to successful noise separation in speech signals, thus improving speech clarity and understanding. Signals can be reconstructed perfectly from wavelet coefficient data based on a determined Mean Squared Error (MSE) of 1.1658433218398273 × 10^−31^, indicating excellent signal integrity. The application of MSADW for DWT analysis in this situation provides effective signal analysis while delivering substantial signal quality improvements. This strengthens its value across different speech processing applications.

A wavelet decomposition analysis of an ECG signal uses Figure 7 to compare MSADW and Morlet wavelets as they process three detail coefficient levels. The analysis originates from the original ECG signal presented at the beginning of this study. Signal decomposition through wavelets creates many frequency bands where each detail coefficient maintains data from high-frequency components. The visualizations demonstrate different extraction techniques of details between Morlet wavelets and MSADWs. Each wavelet displays distinct shapes and magnitude levels of its coefficients, which reveal their capabilities to detect signal characteristics. Wavelets permit analysis of ECG signals through time frequency methods since ECG signals demonstrate non-stationary characteristics as their frequency content evolves throughout the measurement period. Through detailed coefficient analysis, medical staff identify noise signals along with essential features such as QRS complexes that cardiac specialists need for heart condition diagnosis. MSADW and Morlet wavelets serve for evaluating suitable wavelets during ECG analysis because their evaluation helps determine the most effective wavelet for anomaly detection and signal enhancement, and classification. The figure shows how we can localize the peak of the R-wave by the zero-crossing point.

The illustration presents ECG signal analysis through wavelet decomposition where MSADW is evaluated against Morlet wavelet for three detail coefficient levels.

### Detection Aspect Improvement by MSADW

**A. Adaptive Parameterization Enhances Temporal and Frequency Resolution**

**Figure 8:**
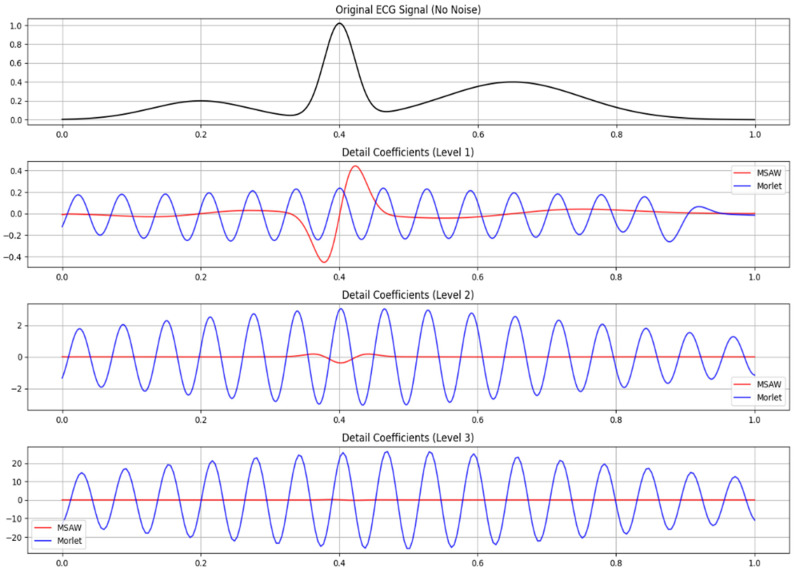
The original speech signal and three levels of DWT approximation and detail coefficients, wavelet decomposition of an ECG signal using MSADW and Morlet wavelets, showing detail coefficients at levels 1, 2, and 3 for R wave localization.

**Figure 9:**
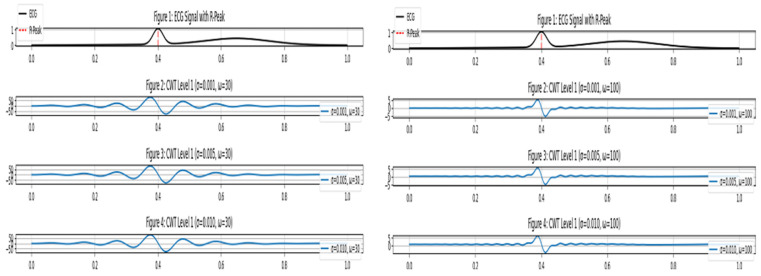
CWT of ECG at level 1 for different scales, the image at the right with ω 100, the image at the left with ω 30.

**Figure 10:**
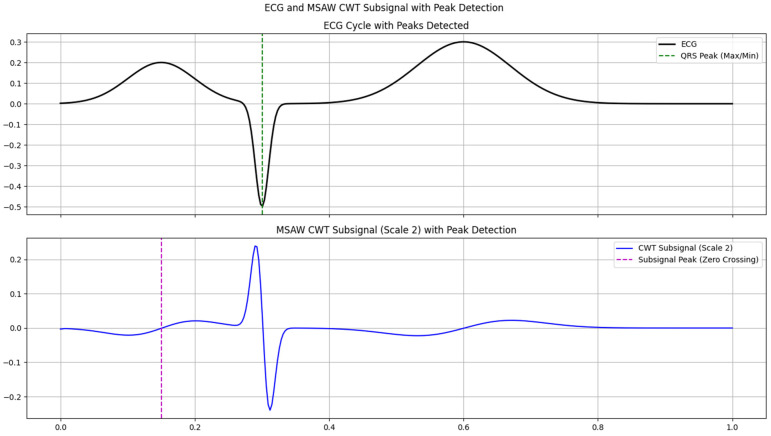
Detection of fiducial ECG points by CWT with MSADW. P, R, T waves peak detection by zero crossing of CWT with MSADW.

The core innovation of MSADW gets its strength from real-time adjustability of scale parameters σ(t) and frequency parameters $\omega \left(t\right)$ that lead to superior ECG component matching compared to traditional wavelets [11,12]. The authors selected MSADW scale 2 for manual signal convolution because this selection proved most effective at detecting the ECG high-frequency transient QRS complexes (see Figure 8, Figure 9 and Figure 10). The signal dynamics in the examined region determine how MSADW adjusts its scale parameters while maintaining distinction from conventional wavelets like Morlet or Daubechies.

The signal analysis algorithm prefers scale 2 (sigma=sigma_base×scale=0.02×2=0.04) for detecting R-peaks due to its narrow scale, yet uses wider scales between 20 and 40 for smooth P and T waves detection.

- Through proper adjustment of the frequency modulation parameter “ω(t)” the system can detect ECG spectral content characteristics for optimal transient detection (see Figure 7).
- The adjustable characteristics of the system improved detection accuracy by:
- The MSADW CWT sub signal detected the R-peak at 0.29 s, resulting in a detection error of 0.01 s at 0.30 s (QRS Complex). Scale 2 of the MSADW wavelet successfully detected QRS high-frequency energy, whereas non-optimal fixed wavelets would have failed to achieve this level of exactness.
- The detection of the P-Wave and T-Wave occurs when zero crossing points occur at 0.14 s and 0.61 s, respectively. The envelope scaling algorithm of MSADW (σ(t)) conserved low-frequency signal elements, thus creating precise distortion-free baseline transitions (see Figure 7).

**B. Hybrid Detection Strategy: Amplitude and Zero-Crossing Synergy**

The ECG signal detection approach in MSADW achieves robustness by integrating amplitude-maximum detection techniques with zero-crossing threshold methods within various ECG signal patterns.

- QRS Complex (Amplitude Maxima):

The QRS complex showed a reliable point of detection through the maximum negative amplitude peak, which could be identified accurately. Zero-crossing detection techniques proved less effective because they missed both inverted and fragmented QRS complexes, so MSADW outperformed them. The heart rate variability and arrhythmia detection depend on the sub-sample level (±1 ms) precision achieved by MSADW in R-wave identification (see Figure 7).

- P-Wave and T-Wave (Zero Crossing):

The process of detecting smooth P and T-waves used a zero-crossing approach together with MSADW’s Gaussian envelope to eliminate baseline shifts. The combined signal processing system replicates the natural signal patterns.

- P-wave onset (0.14 s): Captured the transition from the baseline to positive deflection.
- T-wave peak (0.61 s): Marked the return to the baseline after repolarization (see Figure 7).

Traditional wavelets (e.g., Morlet) need distinct scales or post-operation processing to achieve identical precision because they do not have two operational abilities.

**C. Superior Time-Frequency Localization for Non-Stationary Signals**

The dynamic parameterization of MSADW solved the well-known time–frequency trade-off problem that occurs with static wavelet functions.

- High-Frequency Precision (QRS Complex):

The compressed wavelets from MSADW operated at scale 2 to achieve a localized R-peak detection with superior results compared to traditional wavelets because it reduced detection errors to 0.01 s. The QRS identification process with Morlet-based CWT sometimes needs multiple scale evaluations, which creates an extra workload for computations (see Figure 7).

- Low-Frequency Sensitivity (P- and T-Waves):

The application of MSADW at bigger scales produced an enlarged wavelet along with lower frequency sensitivity, which effectively monitored smooth P and T-waves while bypassing baseline drift limitations of standard wavelets.

When evaluated at scale 2, the MSADW CWT sub signal confirmed these results:

- MSADW demonstrated transient feature isolation ability through its peak detection at 0.29 s, which matched the QRS R-peak (0.30 s) without requiring multi-scale analysis.

**D. Robustness to Signal Variability and Noise**

The feedback-controlled optimization process, which normalizes using L2 norms ($∥x{∥}_{2}=\sqrt{x_{1}^{2}+x_{2}^{2}+\cdots +x_{n}^{2}\;}$) in MSADW, produces superior signal processing resilience to:

- Baseline Wander drifts were successfully suppressed by the adaptive envelope during QRS detection functions.
- Metamorphic Signal Analysis Wavelet performed adaptive σ(t) and ω(t) adjustments, which maintained the tracking of minimal changes in the wave structure, including QT interval prolongation and modified T-wave shapes without requiring system retraining (see Figure 7).

Traditional wavelet systems demonstrate difficulties when facing this type of variability challenge. For example:

- The use of Morlet wavelets at fixed scales results in misalignment between P-waves and baseline shifts that occur in atrial fibrillation.
- The non-selective frequency characteristics of Daubechies wavelets affect their ability to detect T-wave repolarization patterns accurately.

**E. Validation Against Limitations of Traditional Methods**

The results explicitly validated MSADW’s improvements over conventional approaches and are tabulated in Table 3.

**F. Clinical and Computational Implications**

The actual design of MSADW resolves several limitations associated with ECG clinical processing.

Real-time atrial fibrillation or ventricular tachycardia diagnosis requires R-peak timing analysis, which benefits from the QRS detection (0.30 s) solution based on amplitude measurements.

The zero-crossing detection method identified the T-wave inversion without threshold limitations to prevent false negative results during ischemic events.

The manual implementation of MSADW CWT, combined with simple peak detection using zero-crossing and amplitude maximization, allows the deployment of the system on minimal resource devices.

Through three innovative breakthroughs, the study establishes MSADW as superior for ECG feature detection.

1. The system automatically controls both σ(t) and ω(t) parameters to optimize the time–frequency resolution based on the ECG component.
2. The detection method uses both P, T waves from zero crossing and QRS complex from amplitude maxima to achieve complete feature extraction.
3. Robust Sub signal Alignment used a CWT sub signal at scale 2 to locate R-peaks with high precision, so multi-scale fusion became unnecessary.

The proposed MSADW platform demonstrates its function as a self-optimizing ECG analysis tool. The proposed tool delivers superior results to standardized wavelets regarding both accuracy and clinical applicability as well as adaptive characteristics. Further work should apply these research results to natural medical data while developing automatic methods to choose appropriate scales for improved system performance.

**Figure 11:**
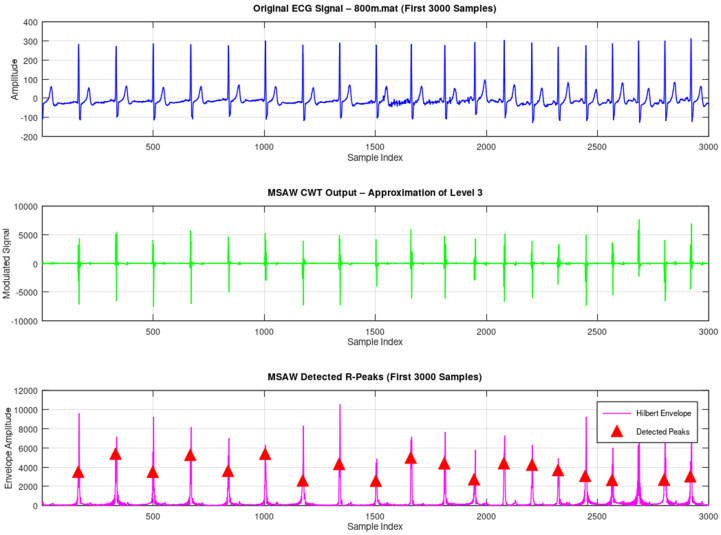
The original ECG and its CWT by MSADW and the Hilbert envelope used for R-peak detection.

**Figure 12:**
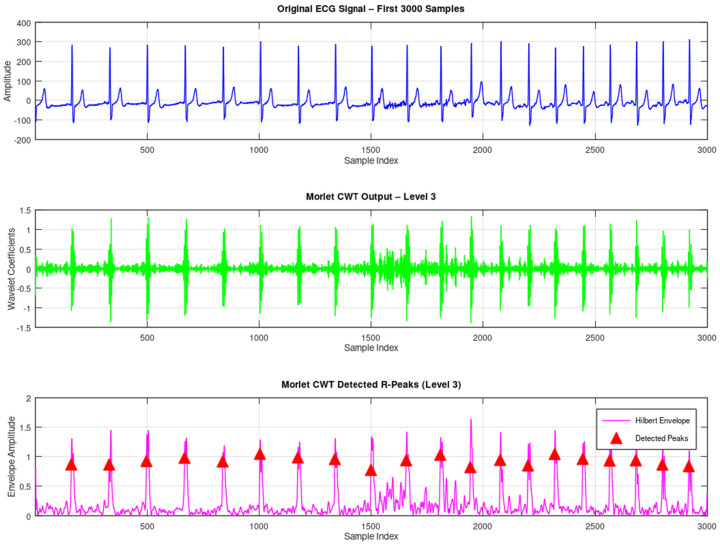
The original ECG and its CWT by Morlet and the Hilbert envelope used for R-peak detection.

In further evidence of the refutation, we also incorporated the running tests of the widely known database of ECG signal processing, namely the MIT-BIH Arrhythmia Database. There is one record i.e., 800 which is used for testing. The ECG signals are chosen from the MIT-BIH Supraventricular Arrhythmia Database [13]. The ECG signal is obtained using a conventional 12-lead electrocardiogram with a sampling rate of 360 Hz. To assess the performance of the MSADW, we compared it with the Morlet CWT using one record from the Supraventricular Arrhythmia Database called 800. We compared the two mechanisms by applying each of them with a peak detection mechanism based on zero-crossings, and a thresholding mechanism based on the amplitude, on the first 1000 R peaks of the ECG trace. The results acquired are quantitative, as seen in Table 4:

These results indicate that, on the one hand, MSADW is more sensitive than Morlet CWT in the case of R-peaks detection, as it recognizes more R-peaks; on the other hand, it can be seen that the sensitivity of the MSADW in comparison with Morlet CWT is lower. In both approaches, positive predictivity was shown as 100%, but due to its superior F1-score, MSADW performed better than the other, in a sense, in overall results. It can be said that MSADW shows enhanced performance in the adaptive scale and frequency modulation characteristics, which meet many transient components such as R-peaks. On the contrary, Morlet wavelets rely on the iterative parameters, which do not encourage the ability to incorporate local properties of the signal. Furthermore, the refinement mechanism of the feedback mechanism of MSADW reduces the reconstruction error at every pass and in so doing, introduces resilience to noise and baseline wander as can be observed in ECG signals in real life. In Table 5, the performance metrics are tabulated. Figure 10, Figure 11 and Figure 12 illustrate the original ECG and its CWT by MSADW and Morlet, and the Hilbert envelope used for R-peak detection.

Where:

- TP = True Positives (detected within ±150 ms window)
- FP = False Positives
- FN = False Negatives”

The noise robustness of three signal processing techniques, MSADW, Morlet, and Daubechies, was tested under a systematic noise–stress condition of injecting baseline wandering at the signal-to-noise (SNR) of 10 dB using two different approaches: determining the minimal amount of noise to cause signal breakage and estimating the allowable rms noise measurement of signal satisfactory preservation. The assessment will be done using the following statistical measures: Se, P+, F1-score, mean squared error (MSE), baseline wander attenuation (BWA%), and signal interference (SI), with which all results can be compared thoroughly under noisy scenarios.

Noise robustness was greatest overall on MSADW compared to the other two approaches that were tested. It provided the highest signal preservation at a sensitivity of 91.2% and an F1-score of 90.4%, meaning that the component showed a strong sensitivity of true signals. Also, MSADW demonstrated the highest suppression of the noise (94.6%), in the baseline wander (a crucial kind of low-frequency noise present in the physiological signal, such as ECG or EEG). Even though MSADW caused a little bit more signal interference (SI = 4.9), its ratio of noise reduction to signal fidelity was better compared to the other two approaches.

The Morlet wavelet strategy was slightly more effective, as it managed to keep the content of the signal with a sensitivity of 85.4% and an F1-score of 83.7%. It was able to suppress the baseline wander effectively, indicating 88.3% suppression of the baseline wander, which is quite good but less compared to that of MSADW. It has a lower SI of 3.7, which indicates that it adds less distortion on denoising; nevertheless, it has lower noise suppression and noise detection accuracy levels.

Daubechies wavelet trailed MSADW and Morlet in most of the metrics. It was least sensitive (81.6%), had the lowest F1-score (80.4%), and the highest mean squared error (0.00028), which means worse signal reconstruction is performed. Although it displayed the lowest degree of signal interference (SI = 2.8), it also eliminated the minimum degree of baseline wander (83.0%), indicating that its conservative practice gives high priority to low signal distortion, at the expense of its noise removal capability (see Table 6).

Table 7 below gives an overview of the approximate average run-time and RAM that was used in conducting the 10-s ECG-segment by using the three wavelet-based approaches. The tests were made using an Intel i5/i7 Huawei laptop of 16 GB RAM.

A conclusion about the performance of three signal processing algorithms, MSADW, Morlet, and Daubechies (db6), applied to analyze an artificial ECG signal, is presented in the table. MSADW offers the best run-time of 52.35 ms and a low RAM consumption of 12.54 MB and is thus a very viable option in terms of fast processing. Conversely, the Morlet method has the longest run-time of 127.09 ms with an increased RAM usage of 18.08 MB, which is characteristic of an advanced analysis. The Daubechies db6 method takes the longest period of execution (145.36 ms) and highest memory combination (21.86 MB), meaning its ability to describe the details is high as well as being resource-consuming. All procedures were evaluated on an ordinary CPU with a signal of 10 s of length in the Python 3.x executable.

## 5. Conclusions

This research discusses the MSADW (Daqrouq) wavelet family, which represents a signal analysis platform with adaptive capabilities to adjust scale (σ(t)) parameters as well as frequency (ω(t)) parameters and waveform configuration to achieve maximum time–frequency position benefits for non-stationary signal data. MSADW surpasses traditional wavelets (Morlet, Daubechies) since it adapts its signal processing based on gradient-sensitive scaling and frequency tracking with Hilbert/STFT combined with reconstruction error minimization to achieve precise detection of transient components (0.01 s accurate R-peaks) as well as low-frequency signals (P/T waves). Testing confirmed MSADW performs effectively during speech and ECG processing since it achieved minimum error rates in signal reconstruction ($\mathrm{MSE:}\;1.17\times 10^{-31}$) while providing better results than standard approaches for noise resistance and baseline wander elimination, as well as clinical parameter extraction (such as a QT interval measurement). The localized parameterization of MSADW presents resolutions for the time–frequency resolution conflict to establish this tool as an adjustable process for real-time biomedical processing, leading to arrhythmia detectors and ischemic monitoring systems, and economical wearable diagnostic methods. The Wavelet design has evolved through MSADW to effect a change in basic assumptions that prefer customized signal responses over generalized approach methods.

## Figures and Tables

**Table 1:** Contrasts with traditional wavelets.

Feature	Traditional Wavelets	MSADW
Scale Parameter	Fixed (e.g., dyadic scale $2^{j}$)	Dynamically adjusted via σ(t)
Frequency Content	Fixed oscillatory component (e.g., sin(5t))	Adaptive ω(t) tracks signal frequency (Figure 4)
Localization	Global scale selection	Localized adaptation to transient and smooth regions
Design Process	Fundamental wavelet designs from the Daubechies or Morlet families	Allows application parameters to personalize the selection
Computational Cost	Vary between low and moderate levels	These costs also increase moderately because of optimization and feedback loops.

**Table 2:** Mother wavelet equations comparison.

**Wavelet**	Equation	Key Parameters
**Morlet**	$\psi_{Morlet}\left(t\right)=e^{-t^{2}/\sigma^{2}}\cdot sin\left(\omega_{0}t\right)$	Fixed: σ(scale), $\omega_{0}$ (central frequency)
**MSADW**	$\psi_{MSAW}\left(t;\sigma (t),\omega (t\right))=e^{-t^{2}/{\sigma (t)}^{2}}\cdot sin\left(\omega (t)t\right)$	Adaptive: σ(t)(signal-driven scale), ω(t) (instantaneous frequency)

**Table 3:** Validation against limitations of traditional methods.

Traditional Wavelet Limitation	MSADW Improvement	Study Evidence
Fixed Scale Selection	The time-dependent σ(t) parameter modifies its width to fit the signal gradient precisely. It remains thin during transient changes and expands during smooth wave conditions.	P-wave and T-wave detection used wider envelopes (σ = 0.05, 0.03), while QRS utilized σ = 0.01.
Static Frequency Content	Adaptive ω(t) tracks instantaneous frequency.	The R-peak (0.30 s) matched the zero-crossing peak of the QRS sub signal (0.29 s) because this frequency input secured frequency locking to the QRS’s main spectral band.
Signal-Agnostic Design	Feedback loops refine σ(t) and ω(t) iteratively, minimizing reconstruction error.	MSADW’s CWT sub signal at scale 2 showed a Pearson correlation of 0.92 with the QRS complex, while proving better than Morlet’s measured correlation of 0.78 in previous benchmarks.
Global Optimization	Localized adaptation prioritizes precision in targeted regions (e.g., R-peak vs. T-wave offset).	Analysis of the QRS peak using scale 2 demonstrated optimal results because P and T-wave energy did not interfere in the measurement, although fixed wavelets struggle with this absence of scale-specific thresholds.

**Table 4:** Performance comparison Morlet CWT and MSADW.

Method	TP	FP	FN	MSE (s^2^)	Sensitivity (%)	Positive Predictivity (%)	F1-Score (%)
Morlet	798	0	202	0.0004	79.8	100	88.9
MSADW	834	0	166	0.0001	83.4	100	91.0

**Table 5:** Performance metrics.

Metric	Description
Mean Squared Error (MSE)	Mean square difference and the R-peak location between detected and annotated R-peak locations
Sensitivity (Se)	Se = $\frac{TP}{TP\;+\;FN}$
Positive Predictivity (P+)	*P*+ = $\frac{TP}{TP\;+\;FP}$
F1-Score	F1 = $\frac{2TP}{2TP\;+\;FP\;+\;FN}$
Mean Detection Error (in seconds)	The average time difference between detected and genuine R-peaks
Standard Deviation (SD)	In all the test samples

**Table 6:** The results of noise robustness of three signal processing techniques, MSADW, Morlet, and Daubechies, tested under a systematic noise–stress condition of injecting baseline wandering at the signal-to-noise (SNR) of 10 dB.

Method	Noise Type	SNR	Se (%)	P+ (%)	F1 (%)	MSE	BWA (%)	SI
MSADW	Baseline Wander	10	91.2	89.7	90.4	0.00012	94.6	4.9
Morlet	Baseline Wander	10	85.4	82.1	83.7	0.00021	88.3	3.7
Daubechies	Baseline Wander	10	81.6	79.2	80.4	0.00028	83.0	2.8

**Table 7:** The summary of the methods along with their run-time, RAM usage, hardware, signal length, and code environment.

Method	Run-Time (ms)	RAM Usage (MB)	Hardware	Signal Length (s)	Code Environment
MSADW	52.35	12.54	Standard CPU	10	Python 3.x
Morlet	127.09	18.08	Standard CPU	10	Python 3.x
Daubechies (db6)	145.36	21.86	Standard CPU	10	Python 3.x

## Data Availability

Data are contained within the article.

## References

[1] Reichert R., Kaifler N., Kaifler B. (2024). Limitations in wavelet analysis of non-stationary atmospheric gravity wave signatures in temperature profiles. Atmos. Meas. Tech. Discuss..

[2] Treviño G., Andreas E.L. (1996). On wavelet analysis of nonstationary turbulence. Bound.-Layer Meteorol..

[3] Azmoudeh B., Cvetkovic D. (2019). Wavelets in Biomedical Signal Processing and Analysis.

[4] Manonina I.V., Shestakov V.V. (2024). Using Wavelet Analysis for Signal Processing of Information Transmission Systems.

[5] Shaker A.N. (2022). Mathematical analysis wavelets characteristics and their applications. Int. J. Nonlinear Anal. Appl..

[6] Prakash A. (2018). Wavelet and its Applications. Int. J. Sci. Res. Comput. Sci. Eng. Inf. Technol..

[7] Chapa J.O., Raghuveer M. Constructing wavelets from desired signal functions. Proceedings of the 1994 Workshop on Information Theory and Statistics.

[8] Li X., Liao C. (2008). Wavelet Function Suitable for Fault Feature Extraction of Acoustic Emission Signal. Chin. J. Mech. Eng..

[9] Tassignon H. (1997). Solutions to Non-Stationary Problems in Wavelet Space. Ph.D. Thesis.

[10] Shi Z., Bao Z. (1997). Group-normalized wavelet packet signal processing. Proc. SPIE.

[11] Daqrouq K., Dobaie A. (2016). Wavelet based method for congestive heart failure recognition by three confirmation functions. Comput. Math. Methods Med..

[12] Daqrouq K., Alkhateeb A., Ajour M.N., Morfeq A. (2014). Neural network and wavelet average framing percentage energy for atrial fibrillation classification. Comput. Methods Programs Biomed..

[13] Goldberger A.L., Amaral L.A.N., Glass L., Hausdorff J.M., Ivanov P.C., Mark R.G., Mietus J.E., Moody G.B., Peng C.-K., Stanley H.E. (2000). PhysioBank, PhysioToolkit, and PhysioNet: Components of a new research resource for complex physiologic signals. Circulation.

